# A case of percutaneous cryoablation complicated by subcapsular hemorrhage managed conservatively

**DOI:** 10.1016/j.radcr.2022.03.061

**Published:** 2022-04-08

**Authors:** Alexandra M. Dubinin, Gerant Rivera-Sanfeliz, Ithaar Derweesh, David Duncan

**Affiliations:** a University of California, San Diego School of Medicine, 9300 Campus Point Dr, La Jolla, CA 92037, USA; b Department of Radiology and Urology, University of California, San Diego Health, La Jolla, CA, USA; c Department of Urology, University of California, San Diego Health, La Jolla, CA, USA; d Department of Radiology, University of California, San Diego Health, La Jolla, CA and Vanderbilt University Medical Center, Nashville, TN, USA

**Keywords:** Cryoablation, Renal cell carcinoma, Subcapsular hematoma, RCC, Renal cell carcinoma

## Abstract

Herein, we report a patient who underwent percutaneous cryoablation for suspected renal cell carcinoma and developed a subcapsular hematoma with numerous pseudoaneurysms and dramatic structural deformity. Despite the severity suggested by the radiologic presentation, a conservative management approach was selected due to the patient's favorable hemodynamic status. This resulted in a positive outcome as alternative treatment options would have resulted in loss of the organ.

## Introduction

Percutaneous cryoablation is an increasingly common nephron-sparing therapeutic for small renal masses due to its reasonable safety profile especially among patients who are not good surgical candidates. Uncommon hemorrhagic complications of this procedure include retroperitoneal hematomas, hematuria, intraparenchymal pseudoaneurysms, and arteriovenous fistulas that are managed with volume support or arterial embolization. We report a patient case involving a presentation of multifocal pseudoaneurysms and subcapsular hematoma, a rare complication of cryoablation, that was successfully treated with conservative measures despite its dramatic radiologic appearance.

## Case presentation

**Fig. 1:**
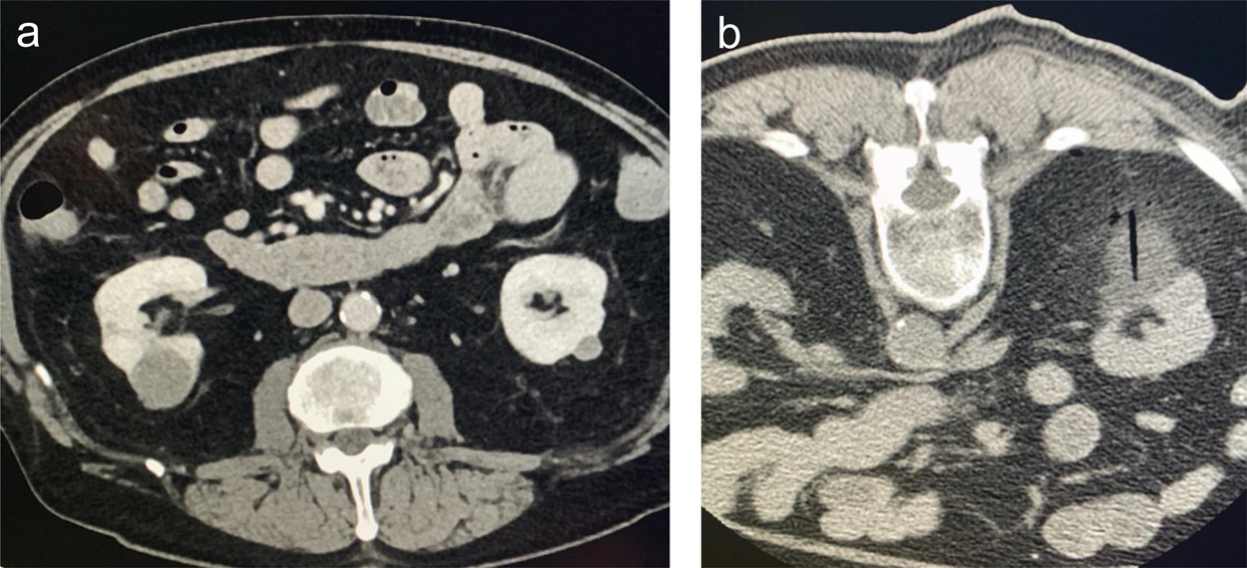
(A) Axial contrast enhanced CT image in nephrographic phase demonstrating a 2.7 cm, homogeneously enhancing renal mass in the right kidney. (B) Cross-sectional image demonstrating cryosection probe in place during second freeze cycle that shows an ice ball covering the treated mass with no significant perirenal hematoma.

A 74-year-old man with past medical history of hypertension, diabetes mellitus, and oropharyngeal carcinoma was referred to the Interventional Radiology department for an incidentally found 2.7 cm, homogeneously enhancing mass in the posterior aspect of the interpolar region of the right kidney, suspicious for a renal cell carcinoma (Fig. 1A). After discussion of the risks and benefits, the patient underwent a CT-guided percutaneous biopsy and cryoablation. Three 18-gauge core biopsies were obtained for pathology. The cryoablation was performed with the insertion of two 0.4 mm cryoprobes under CT guidance followed by a standard freeze thaw protocol: 10-minute freeze, 8-minute passive thaw, 10-minute freeze, 5-minute active thaw. Post-procedural CT scans showed excellent coverage of the mass within the cryoablation zone and no evidence of complications (Fig. 1B). The procedure was considered to be successful, and the patient was admitted to the hospital for routine observation.

**Fig. 2:**
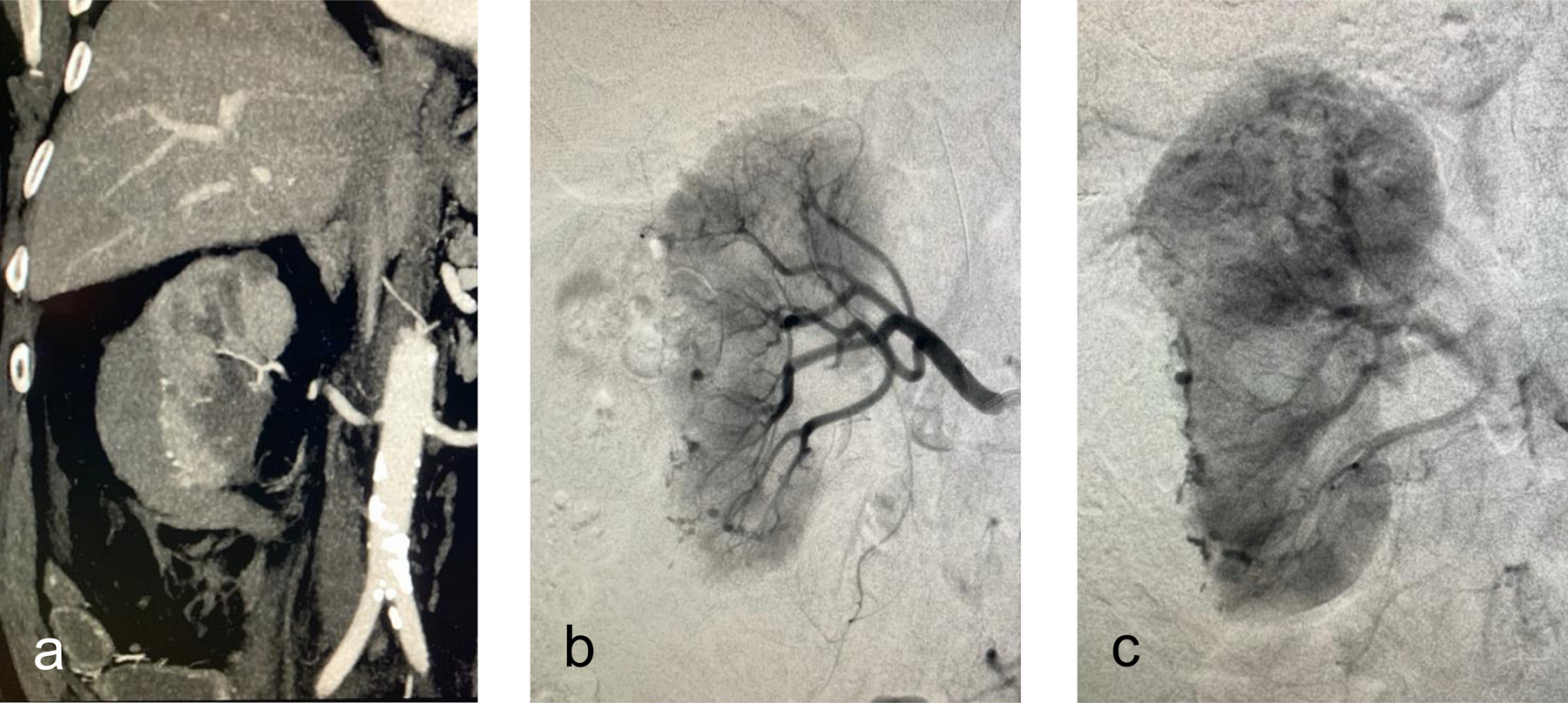
(A) Coronal CT with contrast on post-op day #1 shows a large subcapsular hematoma and renal lacerations associated with multiple pseudoaneurysms. (B) Early arterial phase selective right renal arteriogram demonstrates innumerable subcapsular pseudoaneurysms in the interpolar and lower pole regions, as well as suspicious areas of extravasation in the upper pole region (C) Late arterial phase selective right renal arteriogram confirms areas of extravasation peripherally throughout the entire kidney, some at a considerable distance from the ablation zone.

Over the two days following the procedure, the patient developed a down trending hemoglobin that dropped to 8.2 mg/dL (baseline 11.9). A multiphasic contrast-enhanced CT of the abdomen and pelvis demonstrated a subcapsular hematoma of the right kidney with multifocal active extravasation from the inferior pole of the kidney. Due to the persistent steady decrease in hemoglobin and the imaging findings, the patient underwent emergent renal artery angiography. It revealed numerous pseudoaneurysms arising from the periphery the kidney, most prominent in the mid to lower pole regions and a structural deformity consistent with compression from a large subcapsular hematoma (Fig. 2). An immediate discussion with the urological service regarding the findings ensued. Jointly, the providers decided conservative management was preferred due to the diffuse nature of the injury, which would have required endovascular sacrifice of the entire kidney, and that the hemorrhage appeared to be contained within the renal capsule, likely to self-tamponade. The procedure was terminated, and the patient was monitored closely with serial examinations and laboratories. The patient was hemodynamically stable with a hemoglobin of 8.2-9.4 mg/dL while admitted. His pain was controlled with narcotics. He was discharged three days following the angiographic procedure.

**Fig. 3:**
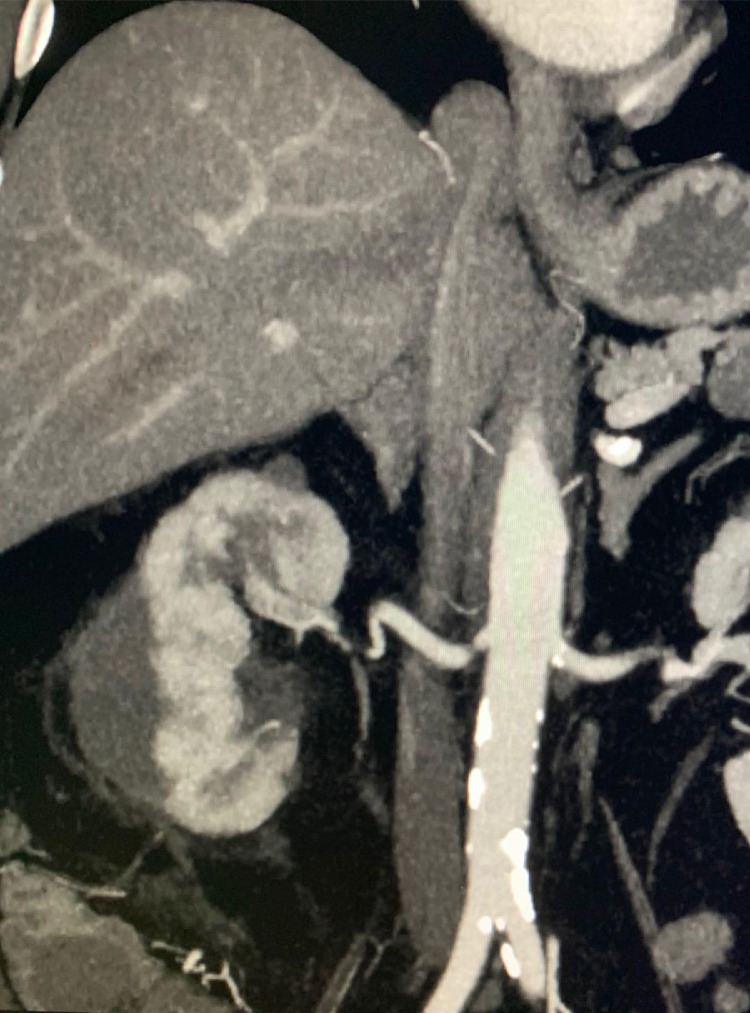
Coronal CT with contrast at 6-week follow-up which demonstrates a decrease in size of the subcapsular hematoma with interval resolution of the pseudoaneurysms.

Active surveillance was initiated per NCCN guidelines. The pathology resulted as papillary renal cell carcinoma, type 1, WHO/ISUP grade 1. A CT performed 6 weeks after the cryoablation (Fig. 3) showed a smaller hematoma with no evidence of active extravasation, pseudoaneurysms, or viable tumor. Imaging at 5 months demonstrated near resolution of the hematoma, no evidence of bleeding, and absence of recurrent or metastatic disease. Creatinine remained essentially unchanged: 1.73 mg/dL before the procedure vs 1.38 mg/dL one year following the procedure.

## Discussion

With the rise in incidence and early detection of renal cell carcinoma (RCC), cryoablation is becoming an increasingly common nephron-sparing therapeutic for clinical T1a (<4 cm) tumors. This method is recommended by the American Urological Association and European Association of Urology as an alternative treatment for high risk T1a RCC patients whose comorbidities make them poor surgical candidates. Cryoablation accounts for 7% of all T1 RCCs treated in the United States [1]. The overall mortality for cryoablation was reported to be comparable with partial nephrectomy, the standard of care first-line therapy for RCCs ≤ 2cm [2]. In select patients, cryoablation may be preferable due to a wide body of literature suggesting superior nephron preservation and a favorable safety profile with cryoablation compared to surgical resection.

In addition to increased utilization, cryoablation applications are being expanded to a broader, surgically ineligible patient population and to larger, less accessible renal masses which has accompanied a recent rise in associated complications. Nonetheless, according to a meta-analysis study, percutaneous procedures still have a significantly lower major complication rate, with 3.1% reported, compared with standard of care surgical procedures at 7.4% [3]. Hemorrhage is the most common adverse event complicating approximately 4.8% of cryoablation procedures [4]. It can manifest as a large retroperitoneal hematoma, hemodynamic disturbances, anemia, hematuria, intraparenchymal pseudoaneurysms, or arteriovenous fistulas. These hemodynamic complications may require interventions such as volume support or even angiography with embolization. Other, less frequent, complications include urothelial injuries, bowel perforation or infection, pneumothorax, and track seeding [5]. Risk factors of complications from cryoablation include advanced age, tumor size, number of cryoprobes necessary, and central location of the mass.

Angioembolization is the standard of care treatment used to mitigate hemorrhages that result in hemodynamic instability that can result from a wide variety of injuries. In this procedure the renal artery or its branches are occluded, permanently or temporarily, through catheter guided injection of an embolic agent. Though minimally invasive, in the case of diffuse renal injury, it caused widespread destruction of the renal parenchyma significantly affecting renal function. Other complications include postembolization syndrome (nausea, vomiting, fever, leukocytosis, and abdominal pain), hematoma, arterial hypertension, and renal failure. To prevent unnecessary complications and preserve renal function the American Urological Association (AUA) guidelines for trauma recommend emergent angioembolization for patients who are hemodynamically unstable despite resuscitation, while those who are stable should receive noninvasive management including monitoring, bed rest, ICU admission, and blood transfusions.

## Conclusion

Despite the large subcapsular hematoma, our patient was hemodynamically stable which guided us to favor conservative treatment over embolization in an attempt to preserve maximum renal function. Because of the widespread nature of the injury, treatment would have resulted in complete loss of kidney function, especially problematic considering his comorbid diabetes mellitus and hypertension. The presented case demonstrates a favorable outcome and provides an example confirming the importance of using hemodynamics to guide clinical decision rather the presence or absence of radiologic derangements.

## Author contributions

All authors provided substantial contributions to manuscript content. All authors gave final approval of the version of the article to be published.

## Patient consent

Patient written and informed consent was obtained for the publication of this case report.
